# Supplementary material for: Evolutionary jumps in bacterial GC content
Source: G3 (Bethesda). 2022 May 17;12(8):jkac108. doi: 10.1093/g3journal/jkac108 (PMC9339322; doi:10.1093/g3journal/jkac108)

**Figure S1. Distribution of scaled phylogenetically independent contrasts in GC content.**


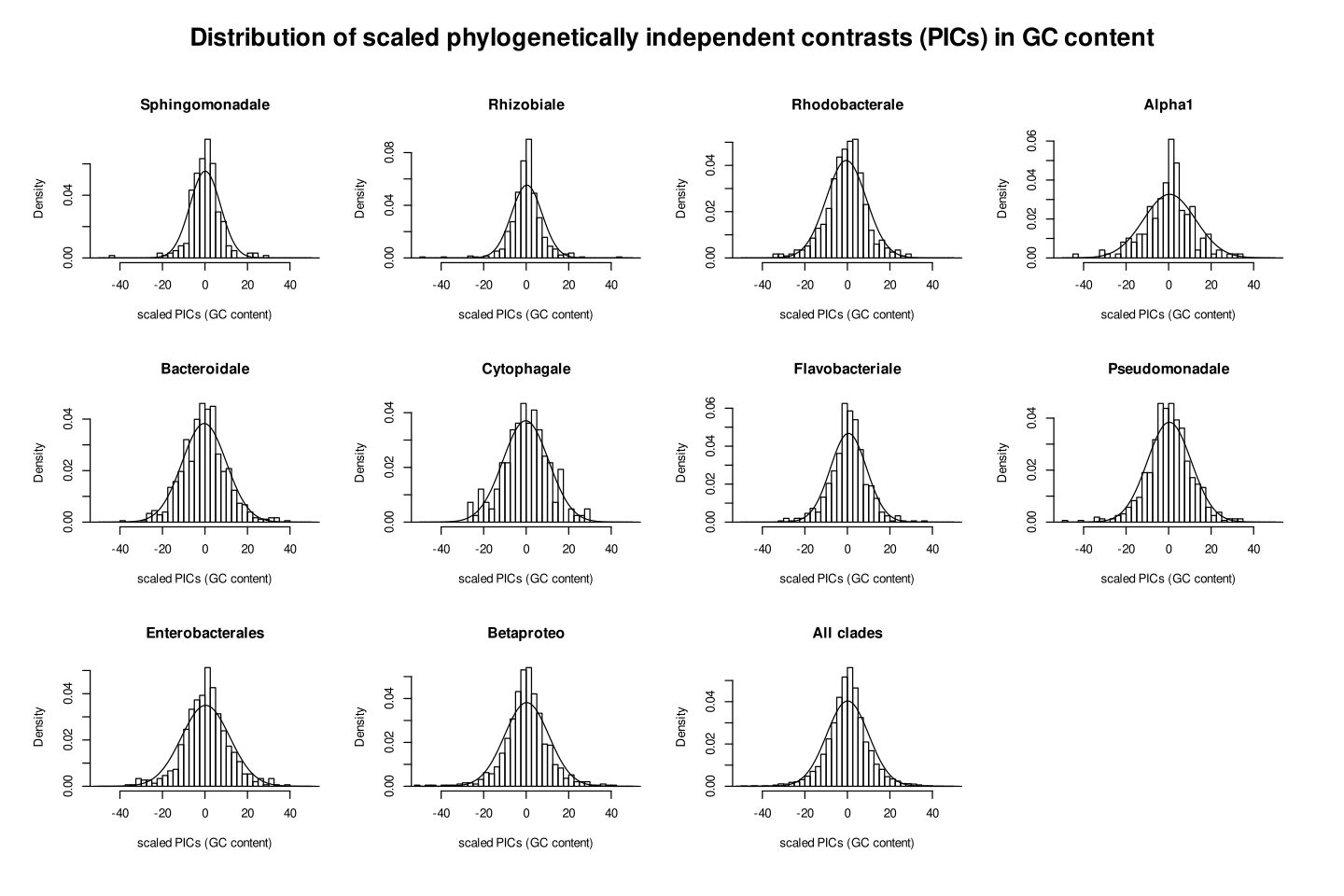
Scaled phylogenetically independent contrasts were calculated using the ape package in R and are plotted as bars. The line represents normal distribution with identical mean and standard deviation.

**Figure S2. The precision-recall relations for jump inference on simulated data.**

Precision and recall of the jump inference procedure was evaluated on 5 simulated datasets for each clade. The orange point and associated label denote the posterior probability threshold chosen to infer jumps in actual data. Alpha1 refers to clade involving Acetobacterales and other related orders of α-proteobacteria.


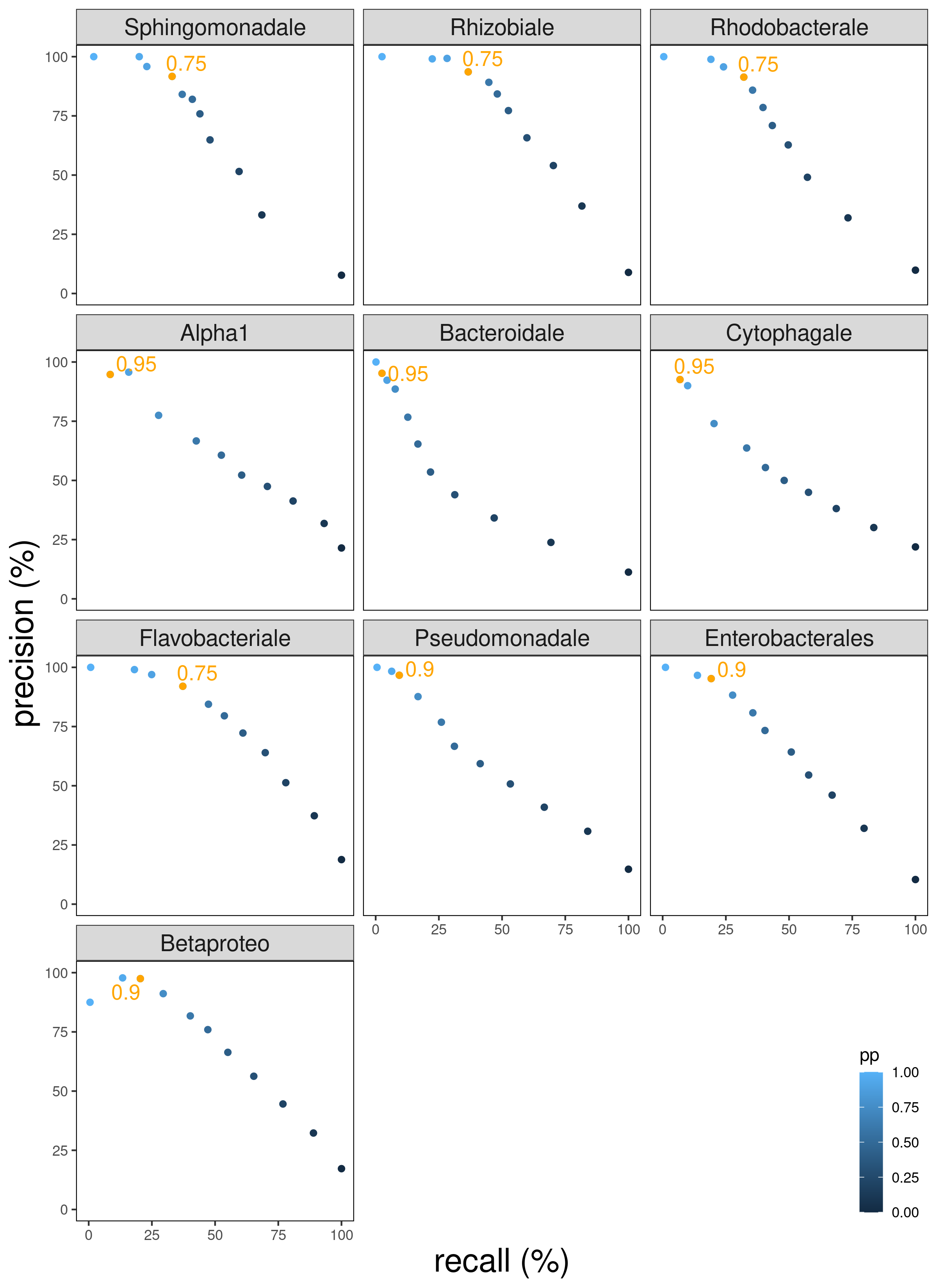


**Figure S3. Recall as a function of jump magnitude in simulated jumps.**

Simulated jumps across all clades and 5 replicate simulations each were pooled (total n~6000) and their magnitudes were obtained as the difference between GC contents of the descendant and ancestral nodes. Jumps were divided according to their magnitude in intervals of 5% change in GC (range -25% to 25%). Fraction of the simulated jumps that were successfully inferred (recalled) according to the procedure in *levolution* were then calculated for each set.


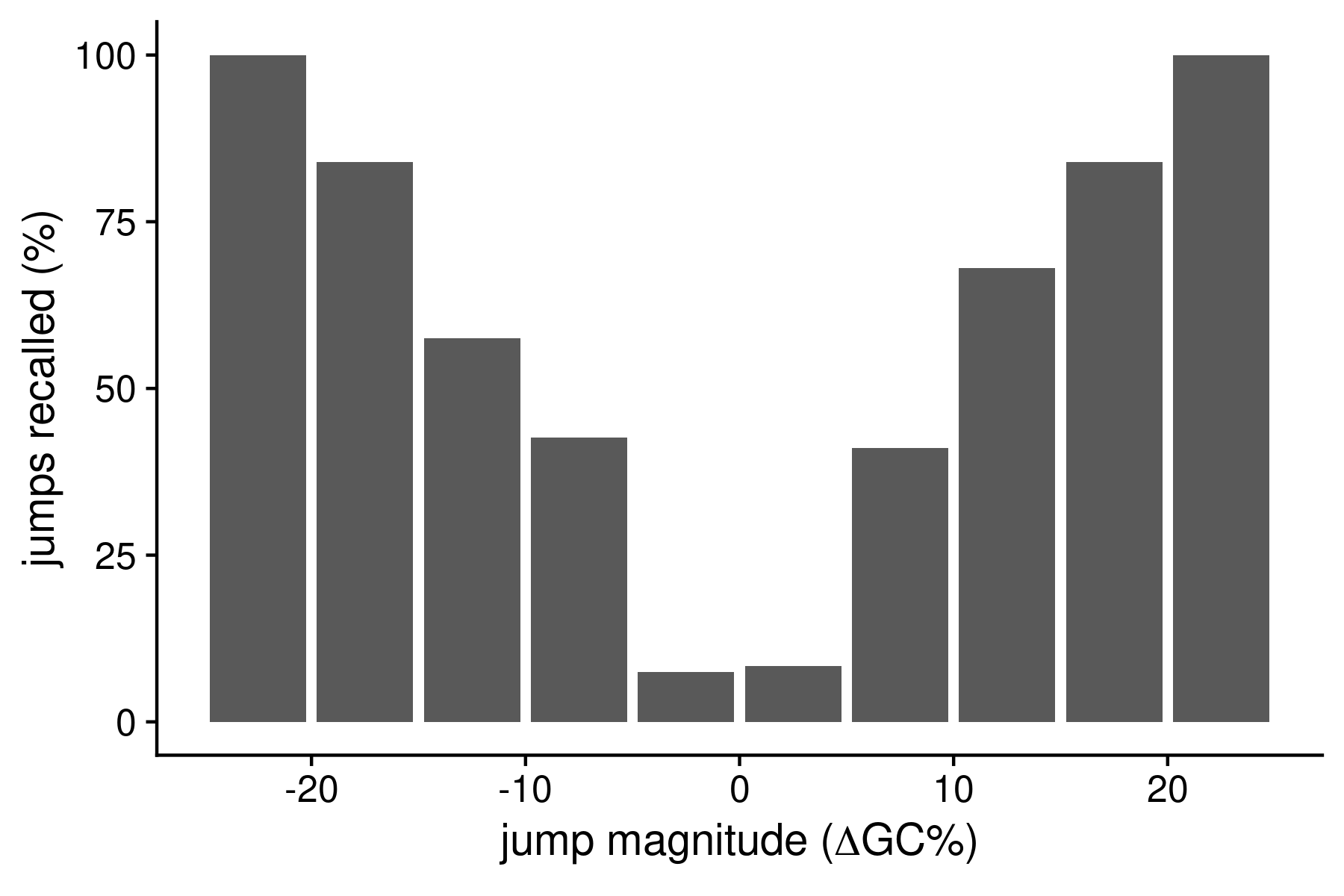
**Figure S4 Distribution of bootstrap support values of nodes**

We extracted bootstrap support values for all nodes (top panel) and nodes that are followed by GC jumps (bottom panel) from the original phylogenetic dataset. A majority of nodes had high bootstrap support values.

**
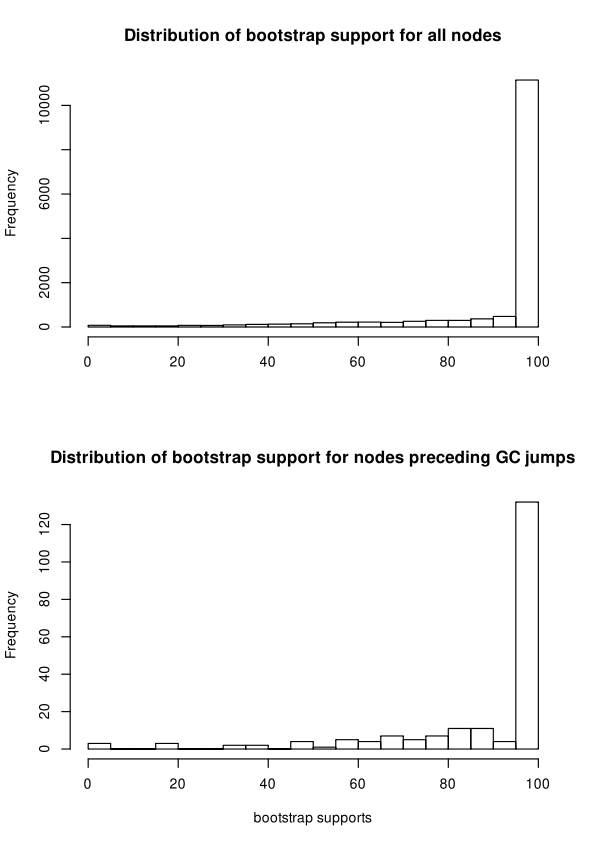
**


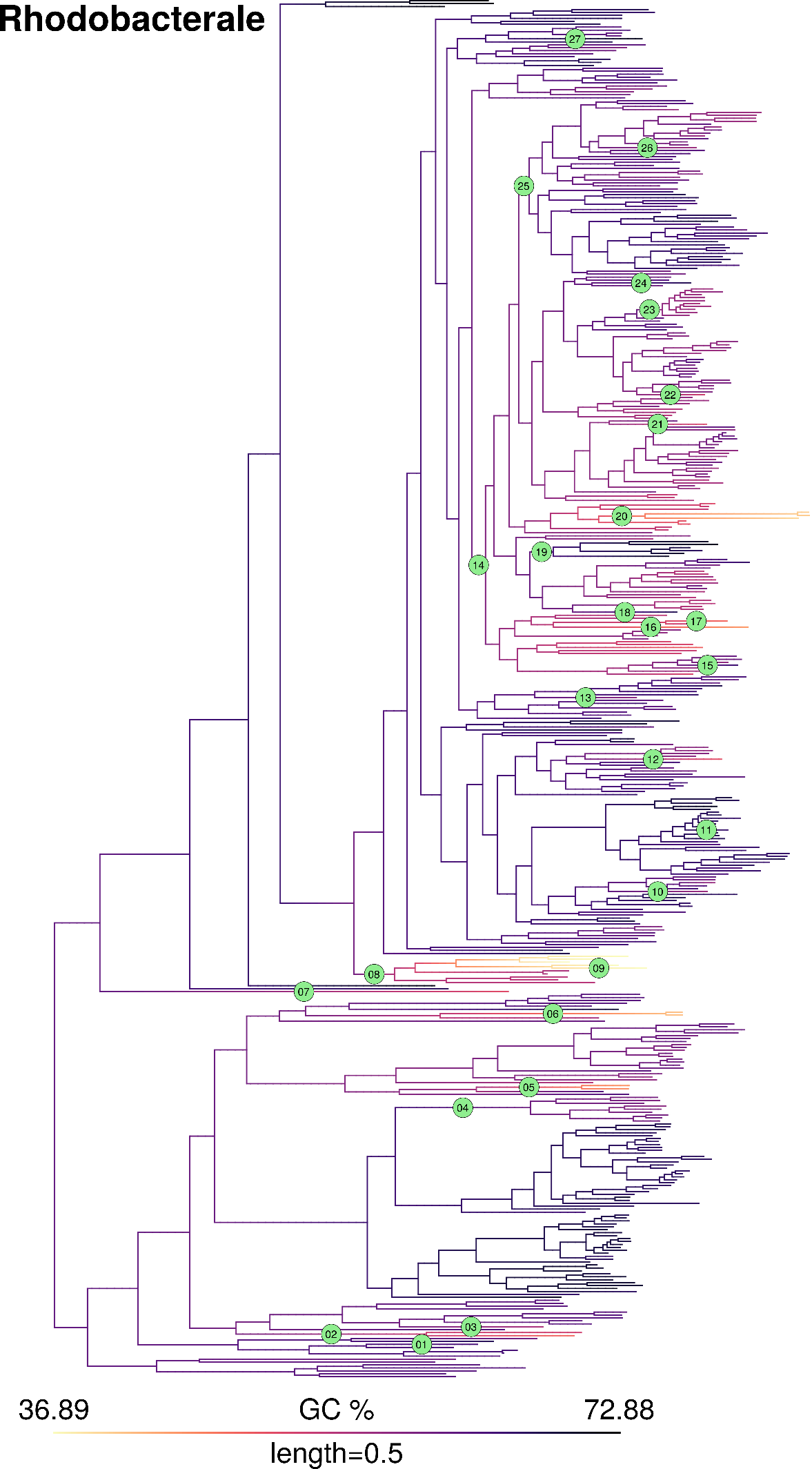
**igure S5 GC content map and location of inferred jumps in order Rhodobacterale.**

**Figure S6 GC content map and location of inferred GC jumps in order Sphingomonadale.**

**
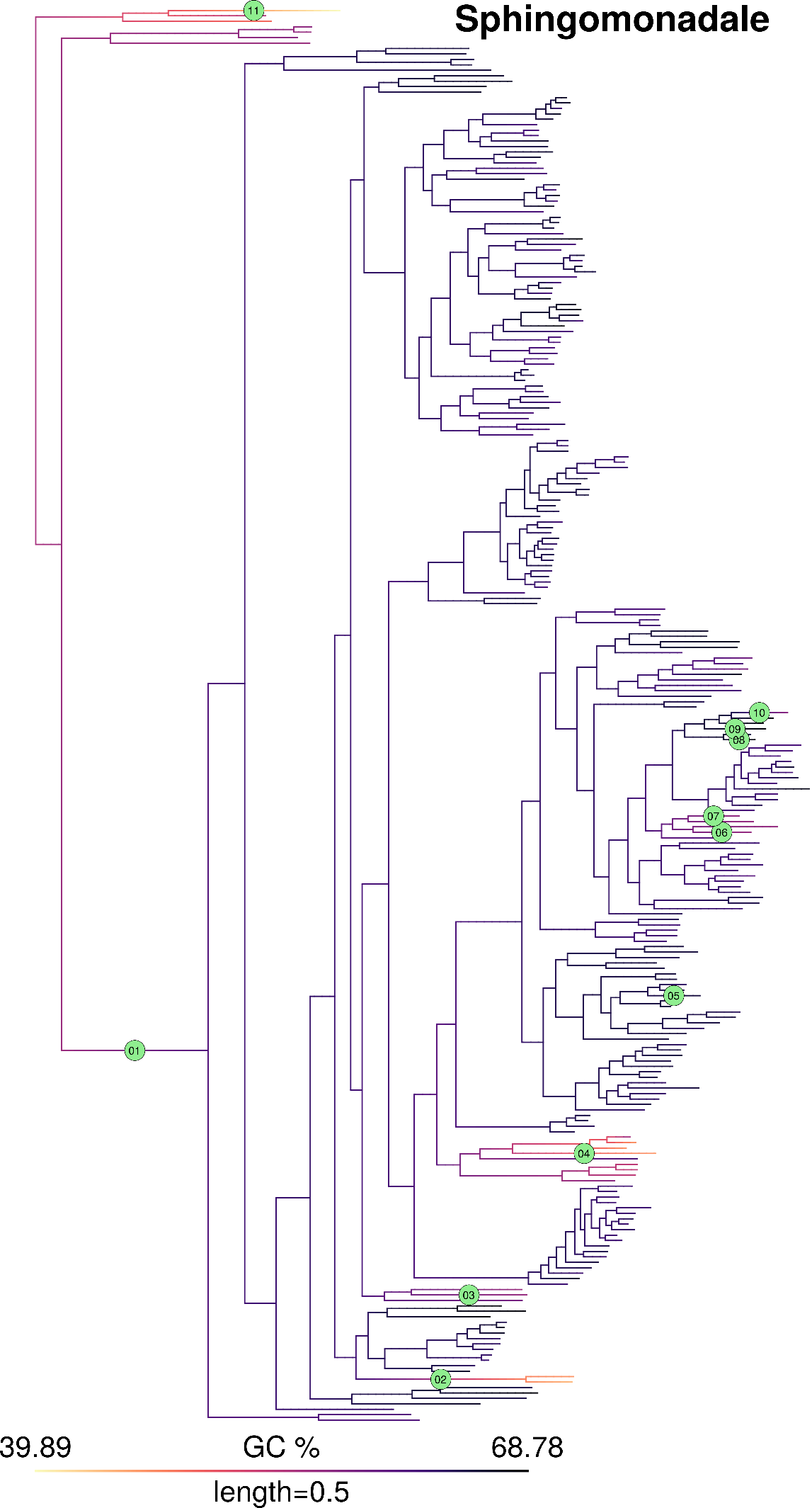
**

**Figure S7 GC content map and location of inferred GC jumps in order Bacteroidale.**

**
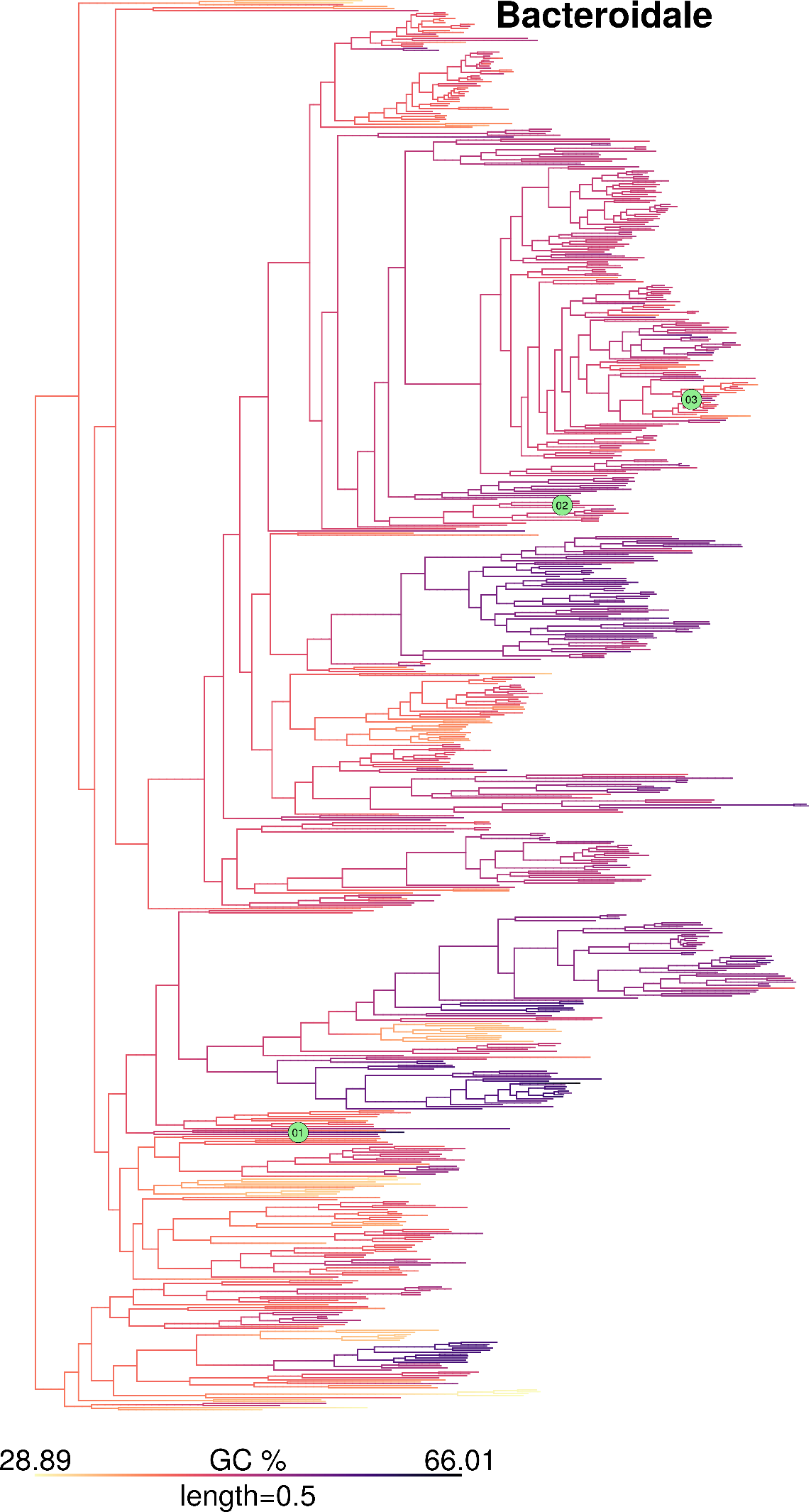
**

**Figure S8. GC content map and location of inferred GC jumps in order Cytophagale.**


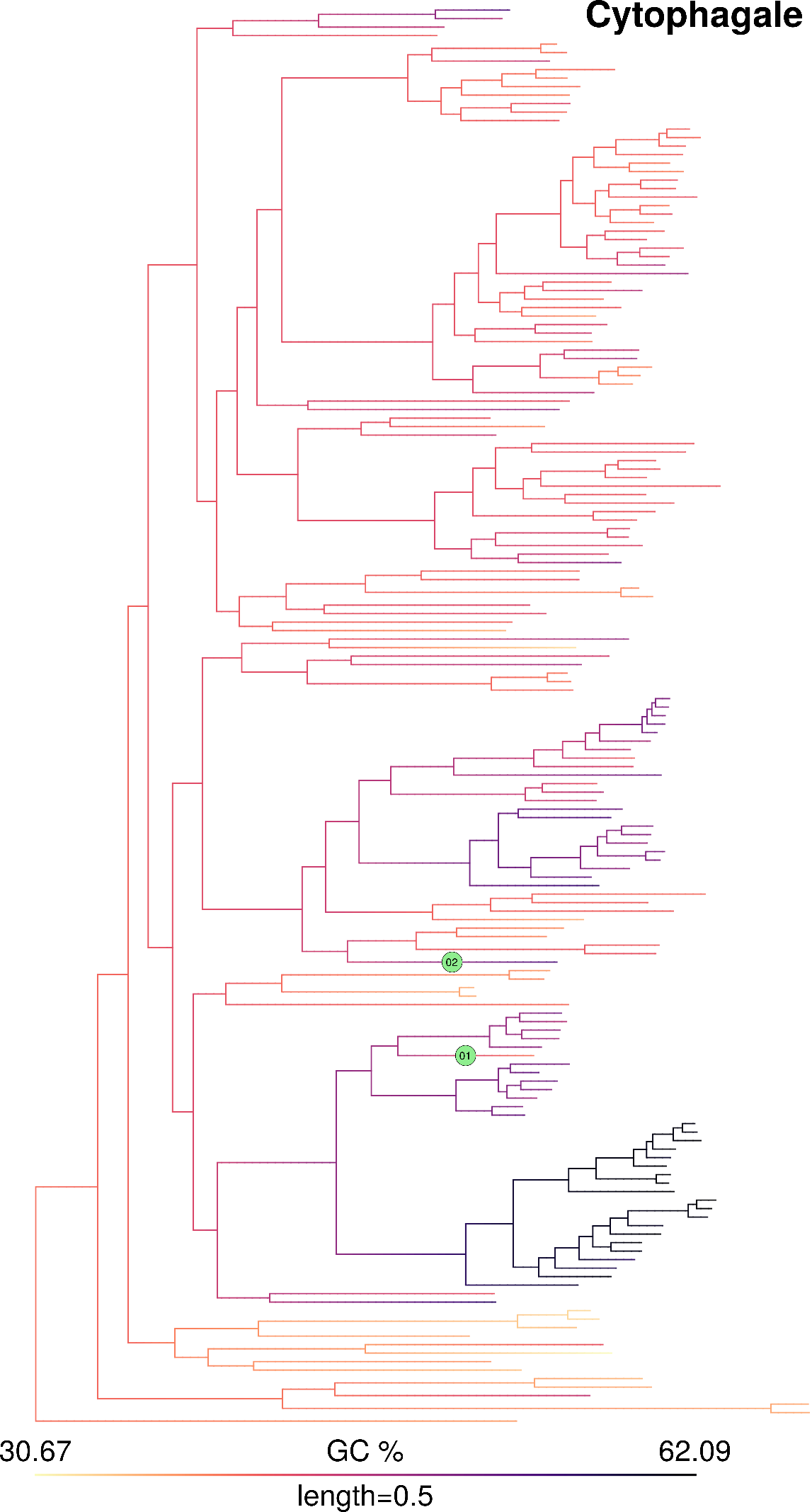


**Figure S9 GC content map and location of inferred GC jumps in order Flavobacteriale.**


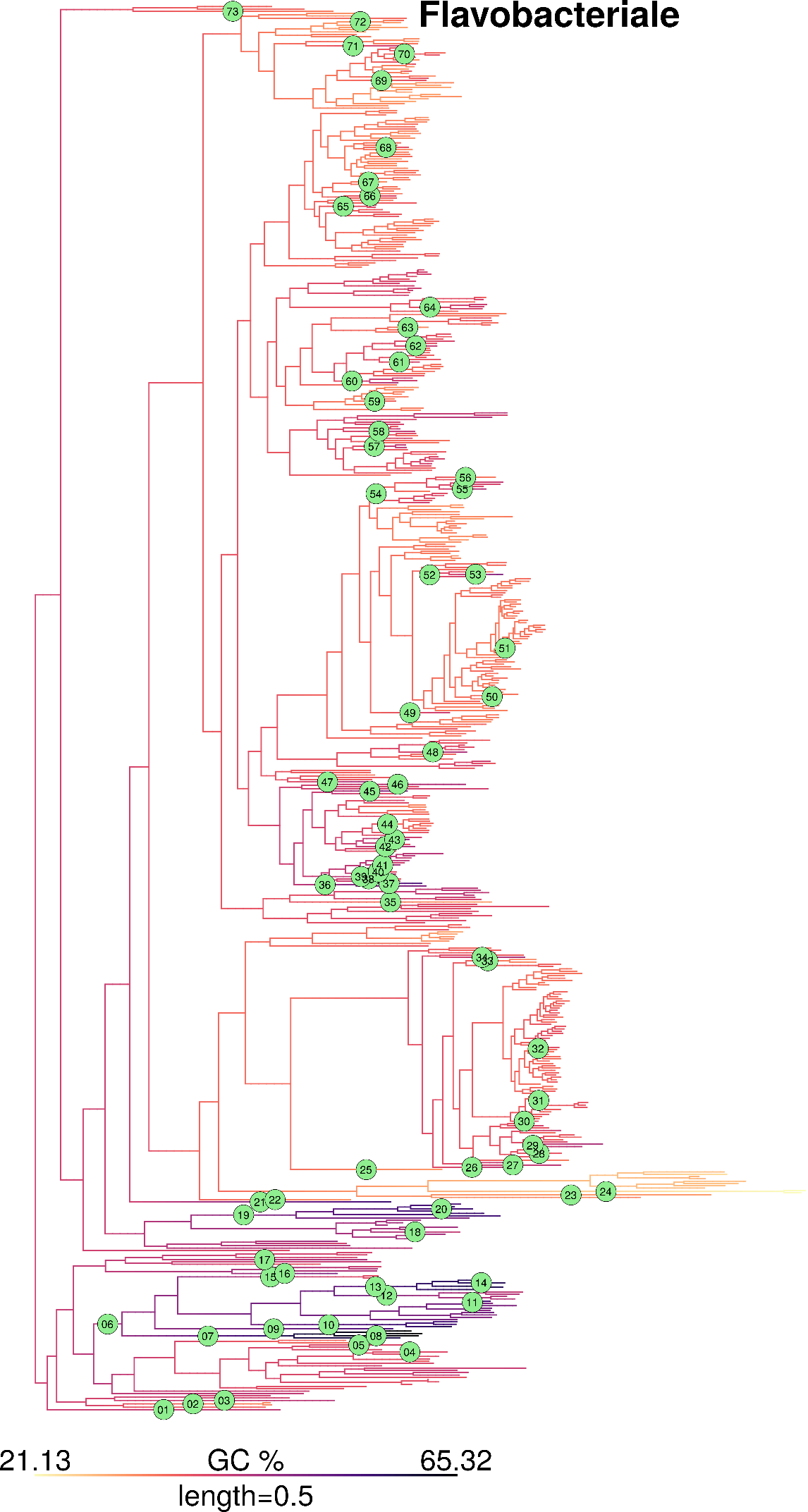


**Figure S10. GC content map and location of inferred GC jumps in order Betaproteobacteriale.**


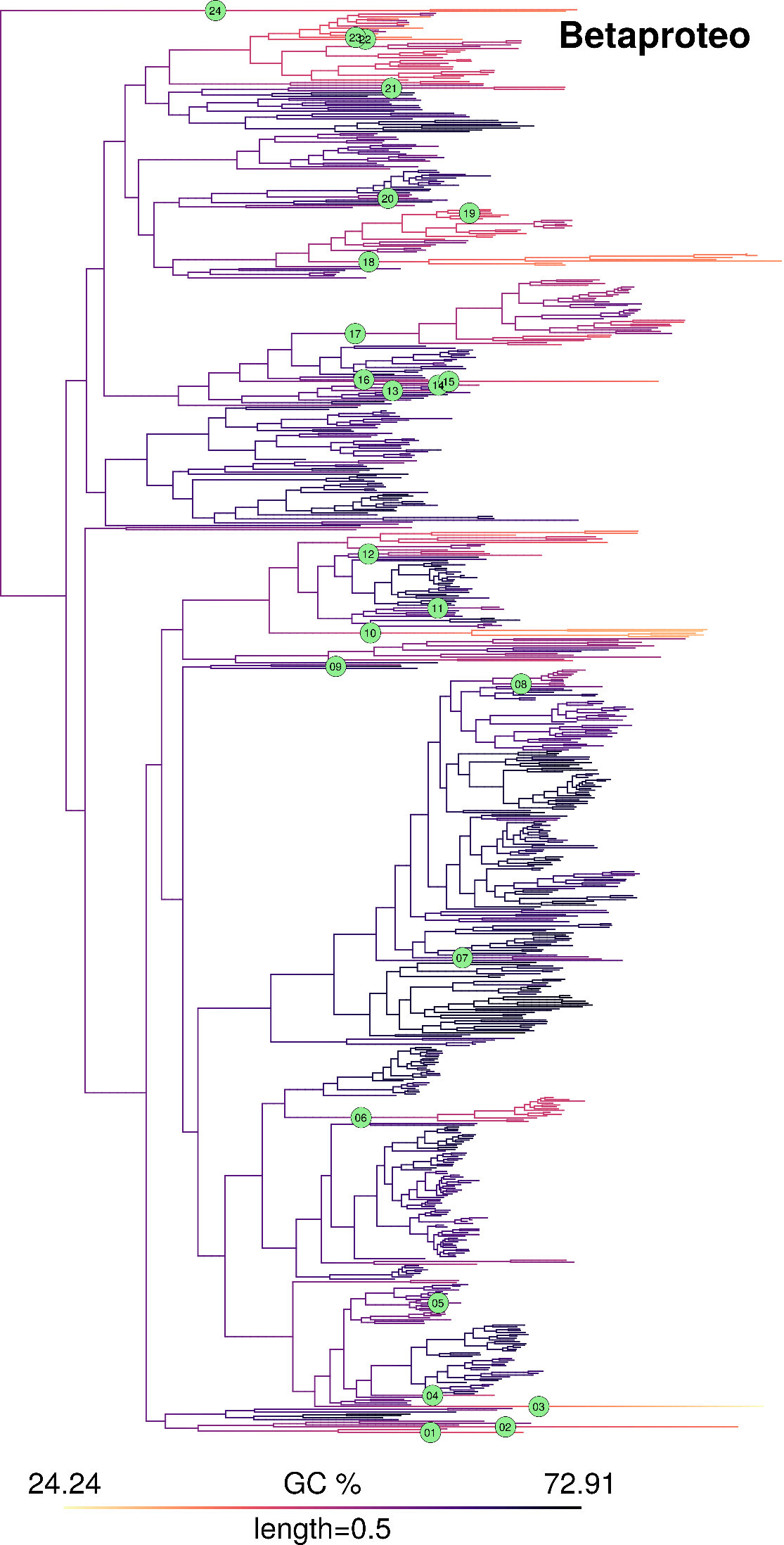


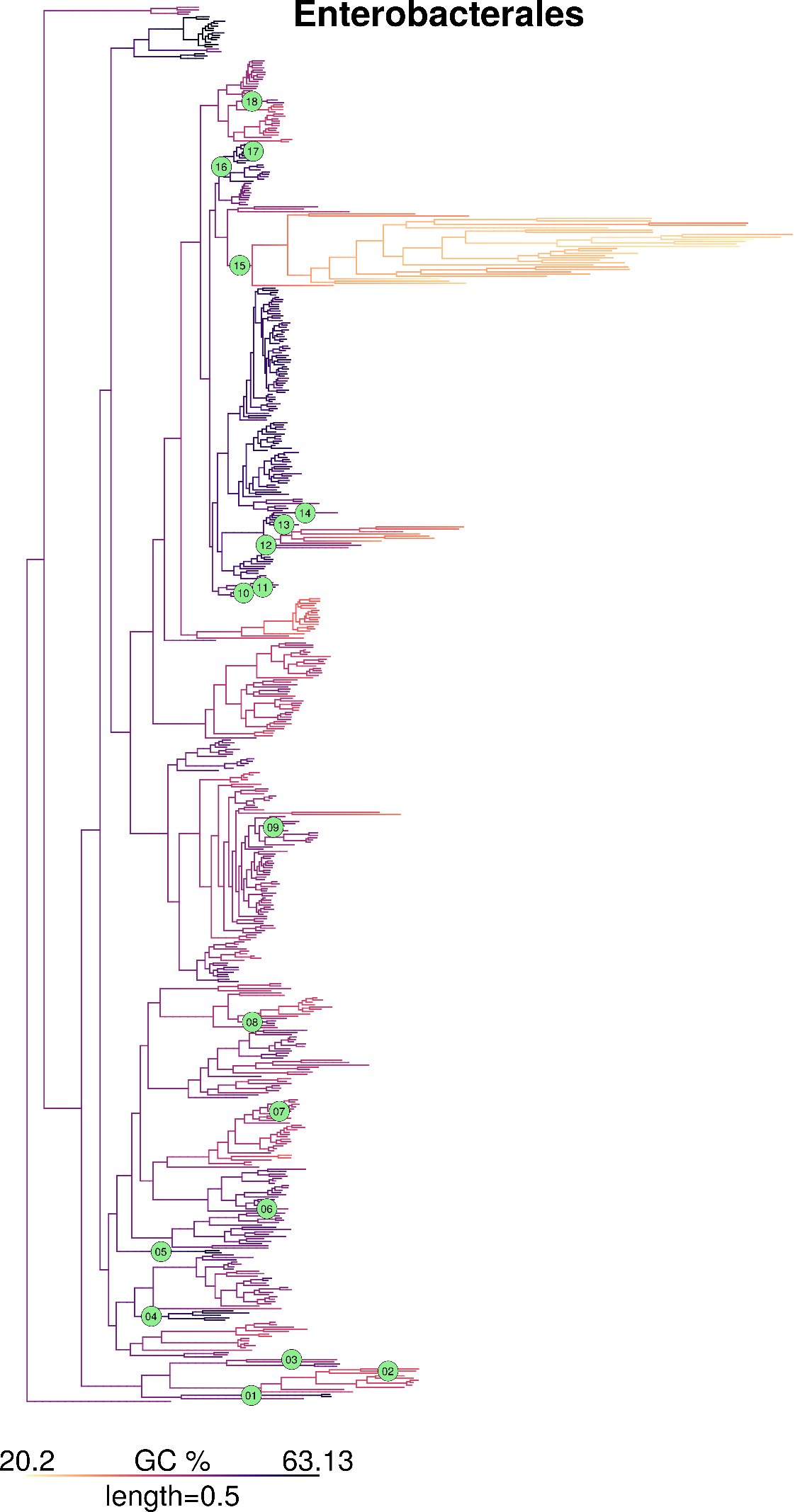
**Figure S11. GC content map and location of inferred GC jumps in order Enterobacterale.**

**Figure S12. GC content map and location of inferred GC jumps in order Pseudomonadale.**


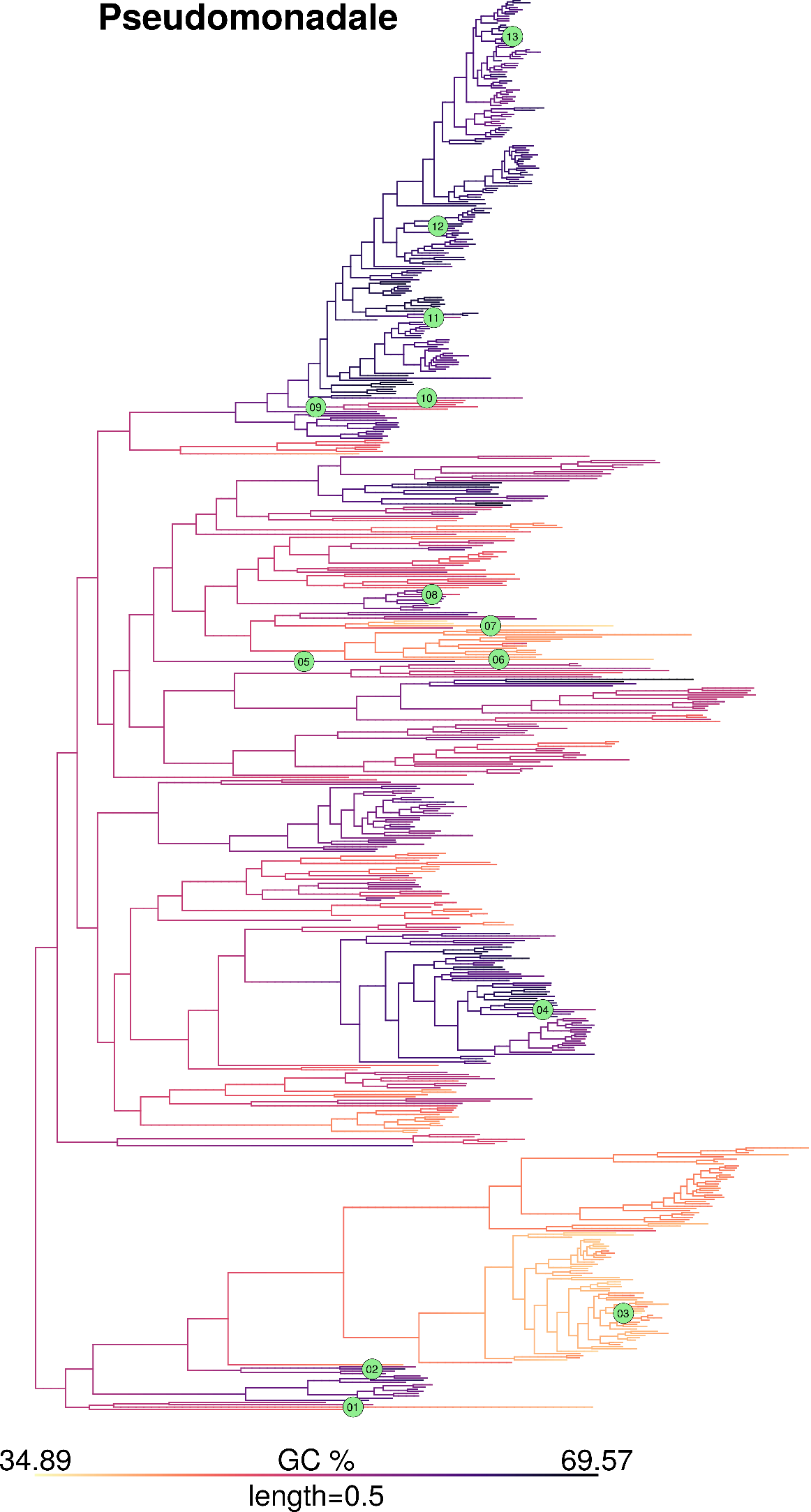


**Figure S13. Distribution of height from root of all branches, randomly placed jumps, and actually inferred jumps**

We compared the distribution of height from root of all branches, heights at which randomly placed jumps are expected, and heights of actually inferred jumps. Height for each branch is represented by the height from the root of the mid-point of the branch. Since the actual location of a GC jump within a branch is not known, we used the height of the branch on which a jump occurred as its proxy. Heights within each phylogeny were normalized by the maximum height and data was pooled across all order-level clades. (a) Distribution of normalized heights of all branches. (b) Distribution of normalized heights of randomly placed jumps. Jumps were randomly placed on branches with probabilities proportional to the branch lengths. Randomly placed jumps were simulated 10 times for each order-level clade. (c) Distribution of normalized heights of actually inferred jumps. The 1^st^ quartile, median, and 3^rd^ quartile positions (shown by dashed orange vertical lines) are not different between randomly placed and actually inferred jumps (Wilcoxon’s rank-sum test, p>>0.05).


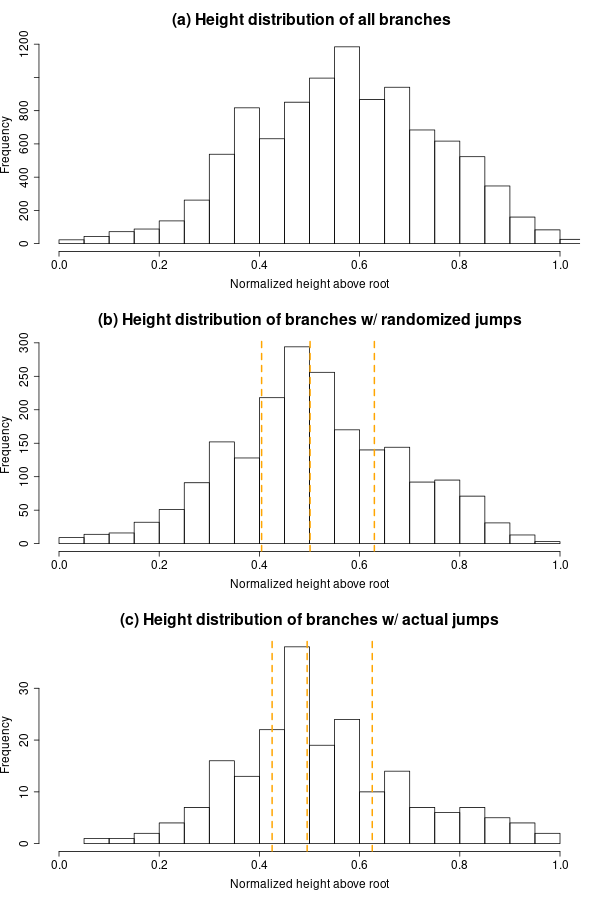


**Figure S14. Changes in GC content of ribosomal protein coding genes are strongly correlated with changes in whole genome GC content.**

We obtained the median GC content of ribosomal protein coding genes in each genome based on the annotations in “cds_from_genomic.fna” files deposited in Refseq. Further, we estimated the changes in GC content in each jump in the same way as described for whole genome GC. Data points are colored based on the magnitude of GC jump given the GC of the whole genome. The blue lines indicate regression lines with 95% confidence intervals. (A) Magnitude of jumps in GC content of ribosomal protein coding genes and genome GC content. The horizontal dashed lines shows no change in GC; whereas the sloped dashed line indicates a regression line with slope 1. The magnitude of GC jumps estimated based on ribosomal protein coding genes are lower, but correlated with those estimated from GC of the whole genome. (B) Median GC content of focal clade genomes based on ribosomal protein coding genes vs. whole genome GC content. (C) Median GC content of sister clade genomes based on ribosomal protein coding genes vs. whole genome GC content.


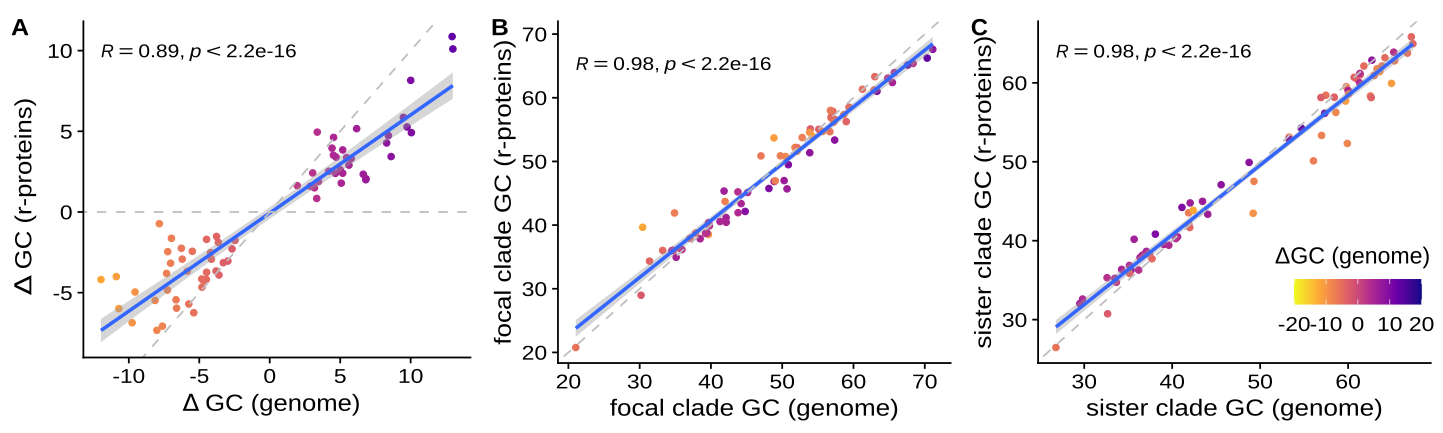

Supplement: jkac108_Supplementary_File_1 [file jkac108_supplementary_file_1.docx]
